# The effects of conventional and self-ligating bracket systems on dental arch changes and tooth movement during levelling and aligning: digital approach

**DOI:** 10.1007/s00784-026-06796-w

**Published:** 2026-02-28

**Authors:** Feyza Nur Simsek, Mucahid Yildirim, Mehmet Esad Güven, Emire Aybuke Erdur

**Affiliations:** 1 Faculty of Dentistry, Department of Orthodontics, Necmettin Erbakan University, Konya, Turkey; 2 Faculty of Dentistry, Department of Prosthodontics, Necmettin Erbakan University, Konya, Turkey; 3 Private Practise, Ankara, Turkey

**Keywords:** Dentoalveolar changes, Digital orthodontic analysis, Tooth angulation and inclination

## Abstract

**Background:**

To compare the effects of Damon Ultima, Damon Q2, and conventional bracket systems on maxillary dentoalveolar structures and the amount of tooth movement.

**Methodology:**

A total of 60 patients (24 males, 36 females) were treated with conventional, Damon Q2, and Damon Ultima brackets (20 patients per group). Intraoral scans were performed with iTero Element™ 2 before treatment, after using round archwires (12 weeks), and after using angular archwires (8 weeks). Arch length, intercanine and intermolar distances, tooth rotation, bucco-lingual inclination (BLI), and angulation changes were analyzed with a 3D accuracy program (Geomagic Control X 2022, 3D Systems, USA). Comparative analyses between the groups were conducted using a one-way ANOVA (*p* < 0.05).

**Results:**

In the intercanine and intermolar distance, significant differences were found (*p* < 0.05); however, in the arc length values, no difference was found between the groups (*p* = 0.270). In BLI and angulation changes, significant differences were observed between the groups. In the angulation movement of the incisor and premolar teeth, there was a significant difference in the Damon Q2 group (*p* = 0.021). In BLI movement of the incisors, there was significantly higher differences in the Damon groups (*p* = 0.001). No difference was observed between the groups (*p* > 0.05) in rotation movement.

**Conclusion:**

Inter-canine and inter-molar distance changes were significant in both Damon systems. BLI and angulation movements of premolars, as well as angulation movements of incisors, were effectively measured. No significant movements were observed in other tooth groups with different brackets.

## Background

The biological response of periodontal tissues to orthodontic forces plays a key role in successful tooth movement and influences the selection of bracket systems and archwires [1]. Friction, a key factor in treatment efficiency, depends on bracket design, archwire material, and the ligation method. To address the issue of friction, self-ligating bracket systems have been developed [2]. Among these, the Damon system (Ormco Corp., USA) has been widely promoted with claims of faster leveling, arch development, and longer intervals between appointments due to reduced friction; however, these claims remain inconclusive according to current evidence [3, 4].

Traditionally, dental arch expansion has been assessed using plaster models [5, 6]. With the advent of digital technology, Standard Triangle Language (STL) scans and dedicated software have enhanced the precision and reproducibility of orthodontic measurements [7, 8]. Despite these advancements, few clinical studies have employed digital tools to analyze tooth movement in detail, particularly angular changes such as rotation, bucco-lingual inclination (BLI), and angulation [9]. Additionally, it remains unclear whether the treatment effects attributed to self-ligating systems are due to the bracket design or the type of archwire employed.

This study aimed to compare the effects of the self-ligating systems (Damon Ultima and Q2) and conventional bracket systems on maxillary dentoalveolar structures and tooth movement rates. The null hypothesis posited that any differences observed in dentoalveolar structures and tooth movement rates among the three bracket systems (Damon Ultima, Damon Q2, and conventional) would not be statistically significant.

## Methods

This study was designed as a prospective observational clinical study. Ethical approval was obtained from the Non-Drug and Medical Device Research Ethics Committee with decision number****dated***. The patient groups were selected from individuals aged 12–25 who applied to the Department of Orthodontics at****University. The sample size was calculated using G*Power 3.1 (Franz Faul, Universität Kiel, Germany) for a two-sample t-test based on the study’s primary outcome, the change in Little’s Irregularity Index after 20 weeks. An effect size of d = 0.45 was selected to represent a clinically meaningful moderate difference in alignment efficiency between the bracket groups. This value was informed by the small effect size (d ≈ 0.21) reported by Ong et al. (2010) in a comparable clinical study comparing bracket types during initial alignment [5]. With α = 0.05 and 80% power, the required sample size was 60 participants (20 per group). Patients were randomly assigned to three groups using Research Randomizer (https://www.randomizer.org/*).* Inclusion criteria for the study are provided in Table 1.

**Table 1:** Inclusion Criteria

Patients with Class I, Class II, or Class III malocclusion without any skeletal discrepancy
Patients with good oral hygiene
Patients with an indication for non-extraction orthodontic treatment
Patients with a midline deviation of less than 2 mm
Patients who have not previously received fixed orthodontic treatment
Patients with moderate maxillary anterior crowding, as assessed by the Little’s Irregularity Index (4–6 mm)
Patients who have not undergone any dental restorations that may affect reference planes during the follow-up period
Patients with a normal growth pattern (SN/GoGn: 32 ± 6)
Patients who have signed the informed consent form

The descriptive results, the scan times, and the wires used are given in Table 2. **Maxillary** intraoral scans were performed with iTero Element™ 2 (Cadent iTero; Cadent Ltd) and STL data were obtained by the same operator (F.N.S). Due to the absence of stable and reproducible reference landmarks in the mandible for longitudinal superimposition, all digital registrations and three-dimensional measurements were restricted to the maxillary arch, which provides a more reliable anatomical lanmarks for accurate superimposition. In all conventional group patients, the ligation method was made by wire ligatures. The archwire was the only active element used; no other auxiliaries were applied during follow-up.

**Table 2:** Demographic Distribution and Used Wires Per Time Intervals

Group	Gender		Age (Years)	Torq Values	Angulation Values	Wires Used	
	Male	Female				T0 to T1 (12 Weeks)	T1 to T2 (8 Weeks)
Conventioanal (MBT 22-inch. Dentaurum Equilibrium. Pforzheim. Germany)	8	12	14.8 ± 3.1	C: +17 L: +10 B: 0 P: -7	C: +4 L: +8 B: +8 P: 0	0.014” CuNiTi	0.014 × 0.025” CuNiTi
Damon Q2 (Ormco. Glendora, Calif.)	7	13	15.2 ± 3.4	C: +12 L: +8 B: +7 P: -11	C: +5 L: +9 B: +5 P: +2	0.014” CuNiTi	0.014 × 0.025” CuNiTi
Damon Ultima (Ormco. Glendora, Calif.)	9	11	15.2 ± 3.8	C: +7 L: +3 B: -2 P: -4	I: +5 L: +9 B: +5 P: +2	0.014” CuNiTi	0.014 × 0.0275” CuNiTi

Demographic Data of the 60 Patients in the Study. Types of Wires Used at Different Time Intervals. All groups used *ORMCO* (Glendora, Calif) wires with Europoform II arch form. I: Centrals, L: Laterals, B: Bicuspid, P: First and second Premolars.

A total of 11 patients (5 from the conventional group, 2 from the Damon Ultima group, and 4 from the Damon Q2 group) were excluded from the study due to inadequate oral hygiene, bracket failure, or missing follow-up appointments. New participants were randomly selected to replace dropouts and maintain study continuity.

### Digital analysis methodology

STL data at the time points were imported into Geomagic Control X 2022 (3D Systems, USA) for analysis. Operator calibration was conducted by an experienced software operator (M.E.G) and an orthodontist (F.S). This process involved repeatedly determining the guide plane and measuring the angular deviation until the measurements of the two operators were found to be similar. The agreement between the measurements was calculated by the ICC (Intraclass Correlation Coefficient) test. The minimum agreement was found to be ICC = 0.976. Subsequently, all analysis procedures were conducted by one researcher (F.S.) to ensure the accuracy of the measurements. The method employed aligns with that described in the study by Papaspyridakos et al. [10].

## Describe the reference planes

Calibrated reference planes were established using unchanged points of the teeth. The global reference axes were defined as follows:

X axis: anterior-posterior direction,

Y axis: left-to-right direction,

Z axis: occluso-gingival direction.

The following planes were then determined on the digital model.

**Fig. 1:**
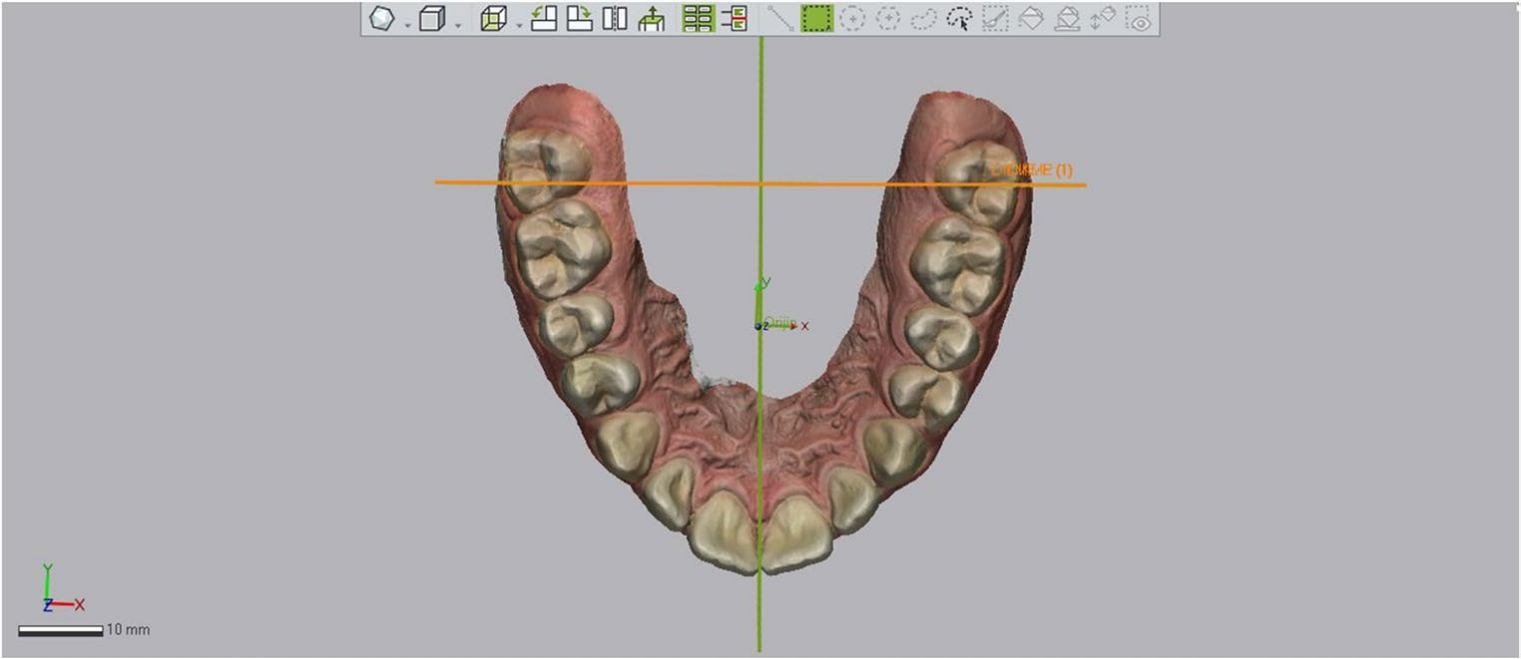
Reference planes used in digital measurements. The MRP, MRP-90, and global coordinate axes (X, Y,Z) are illustrated

The Middle Reference Plane (MRP): Parallel to the X-axis, a plane was defined by selecting reference points that remained unchanged throughout the treatment using the “add plane” tool of the software. These points included the lowest end of the labial frenulum, the crest of the incisive papilla, and the anterior border of the rugae (Fig. 1).

The Perpendicular Plane to MRP (MRP-90): It is defined perpendicular to the MRP in the X plane. It is used only for “angulation measurement” of canine, premolar, and molar teeth, movement in the sagittal direction does not affect the angulation values. (Fig. 1).

**Fig. 2:**
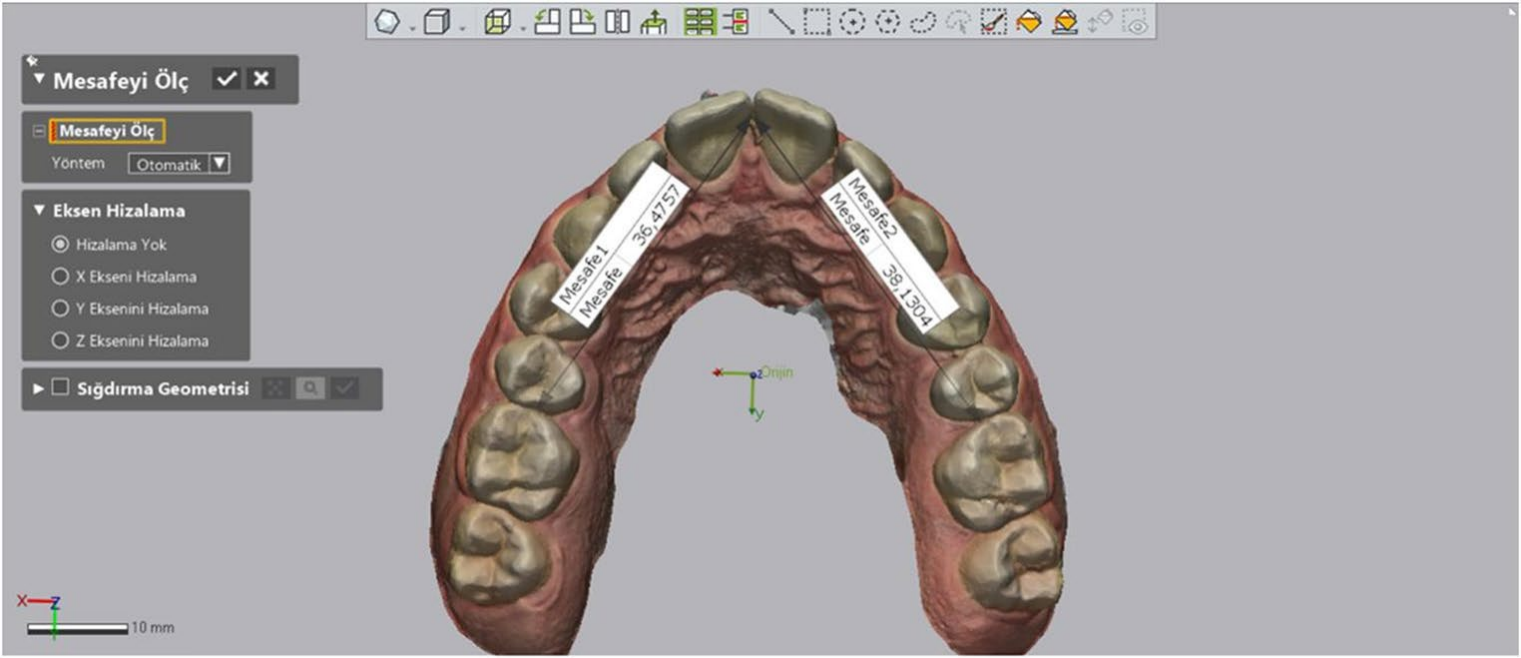
Measurement of arch length

Arch Length: The total length from the anatomical mesial contact points of the first molars to the contact points of the central incisors was measured (Fig. 2).

**Fig. 3:**
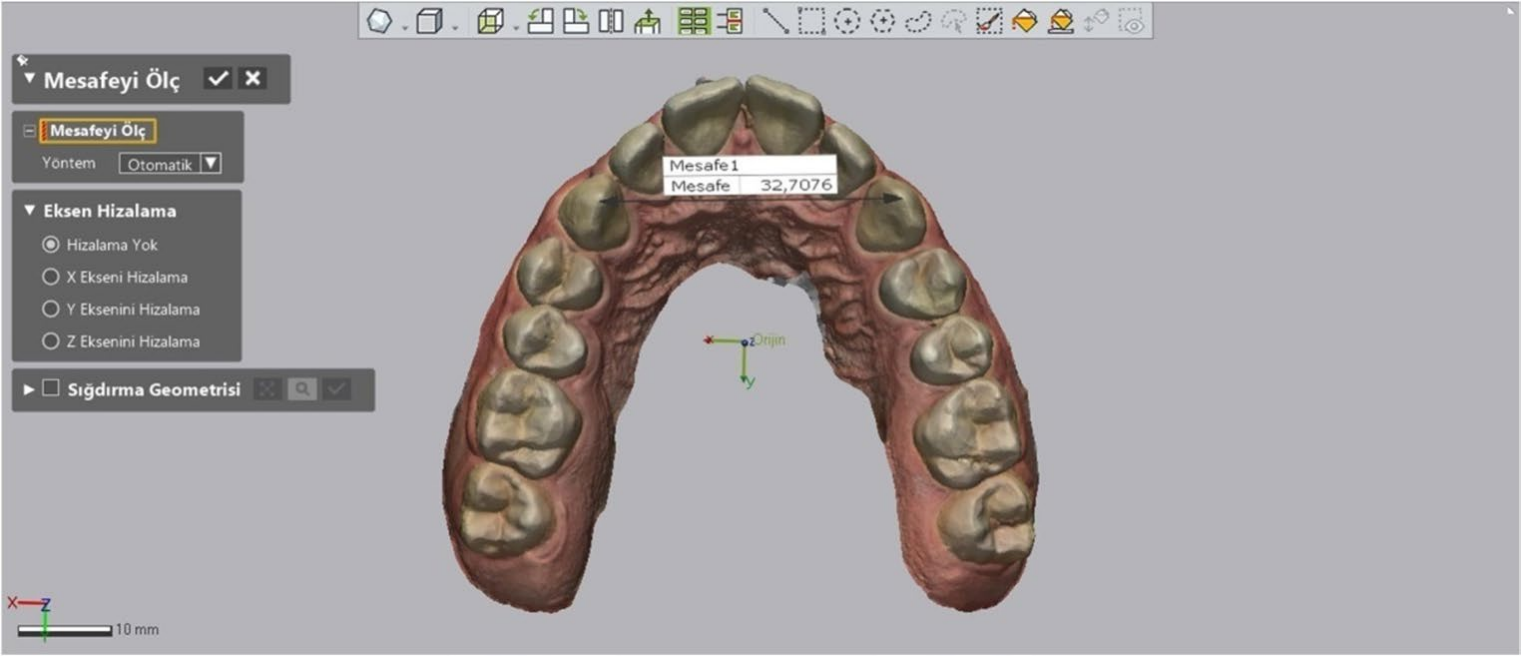
Measurement of intercanine distance

Intercanine Distance: The distances between the canine tips were measured using the software’s “add point” tool and the “linear dimension” tool (Fig. 3).

**Fig. 4:**
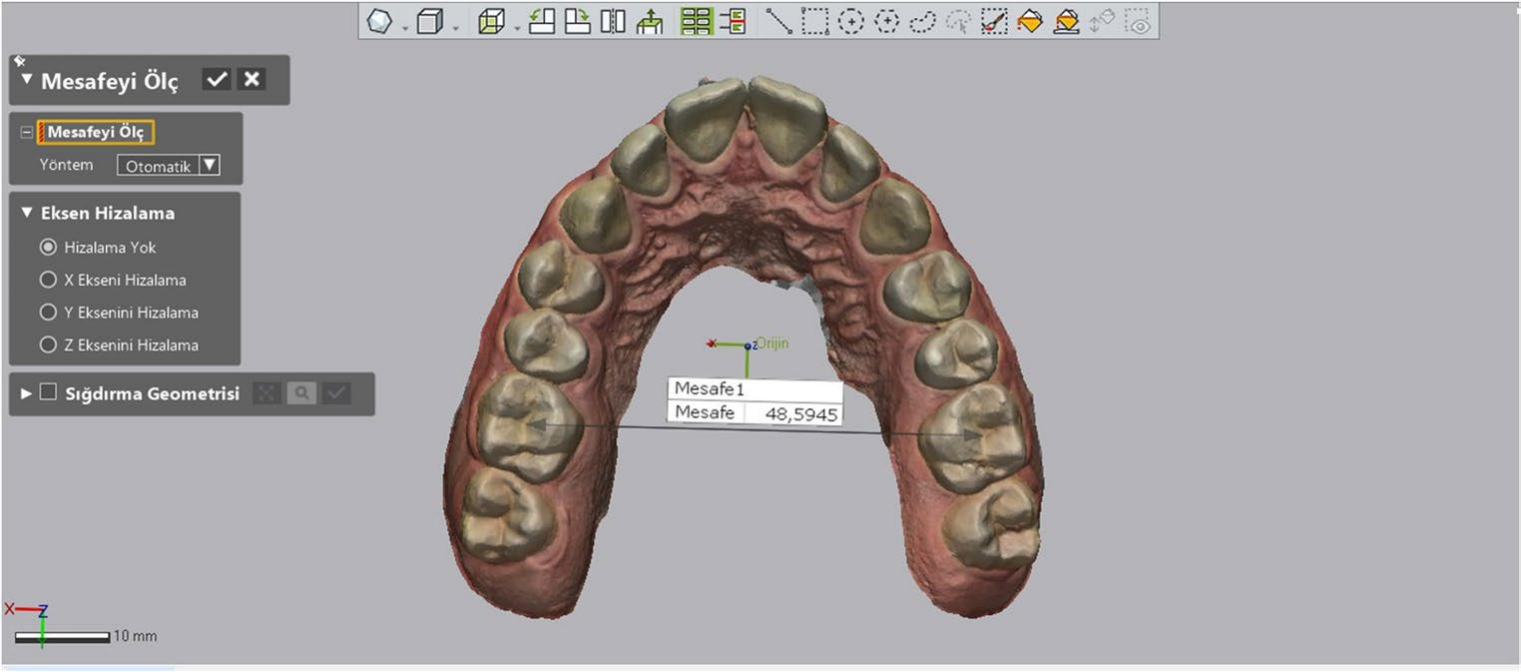
Measurement of intermolar distance

Intermolar Distance: Similarly to intercanine distance, the distances between the central fossae of the molars were measured (Fig. 4).

**Fig. 5:**
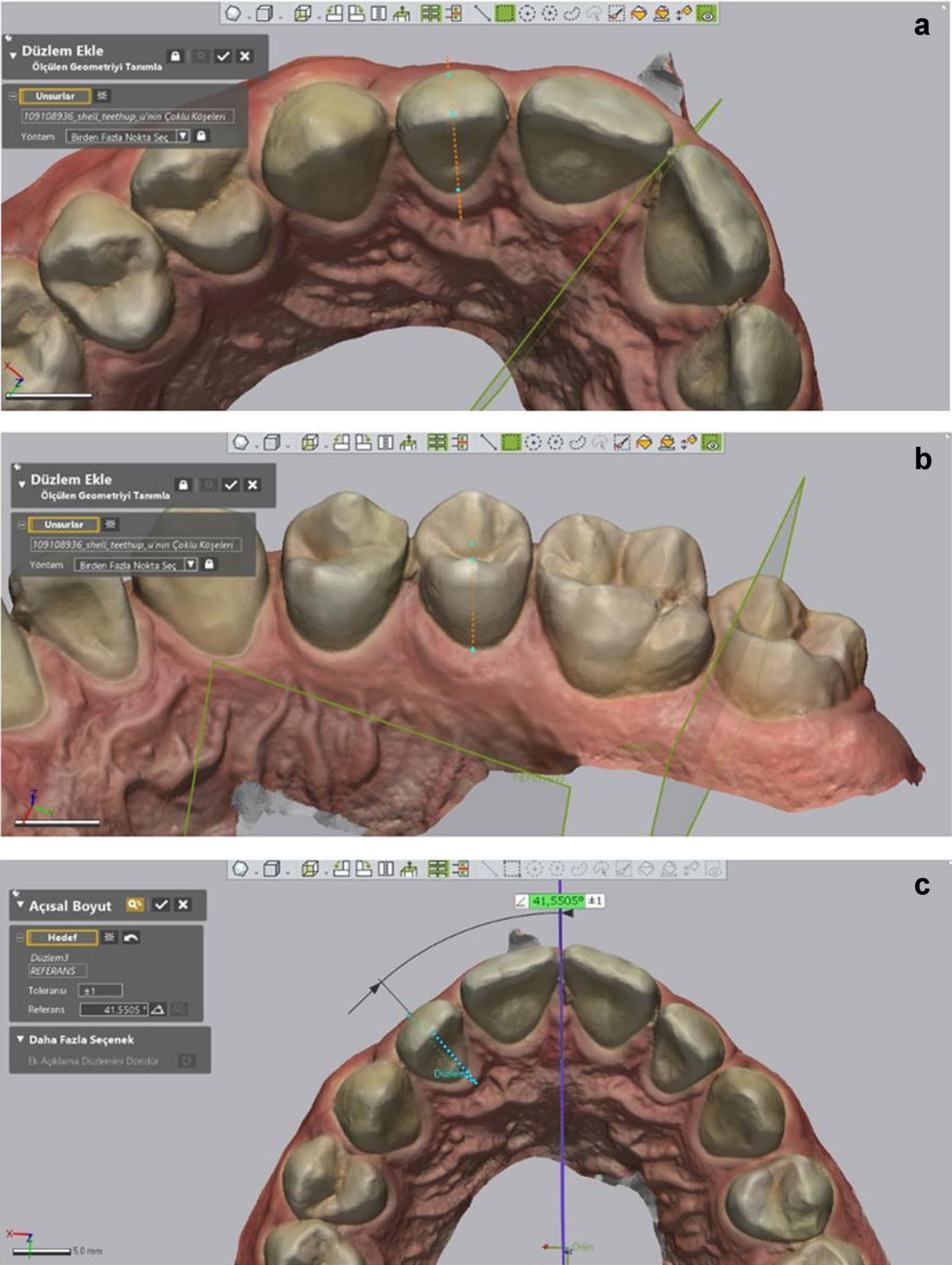
Rotation measurement methodology. (**a**)Reference points of incisor, (**b**)Reference points of premolar, (**c**)Measurement of incisor

**Rotation measurement**: For each incisor, a plane was first defined using the software’s “add plane; pick multiple points” tool. This plane was created by selecting three key points: the deepest mid-buccal point, the incisal midpoint, and the deepest mid-lingual point. For the posterior teeth, a similar plane was defined by selecting the deepest points of the central fossa and mid-lingual areas. The angles between these defined planes and the MRP were then measured (Fig. 5).

**Fig. 6:**
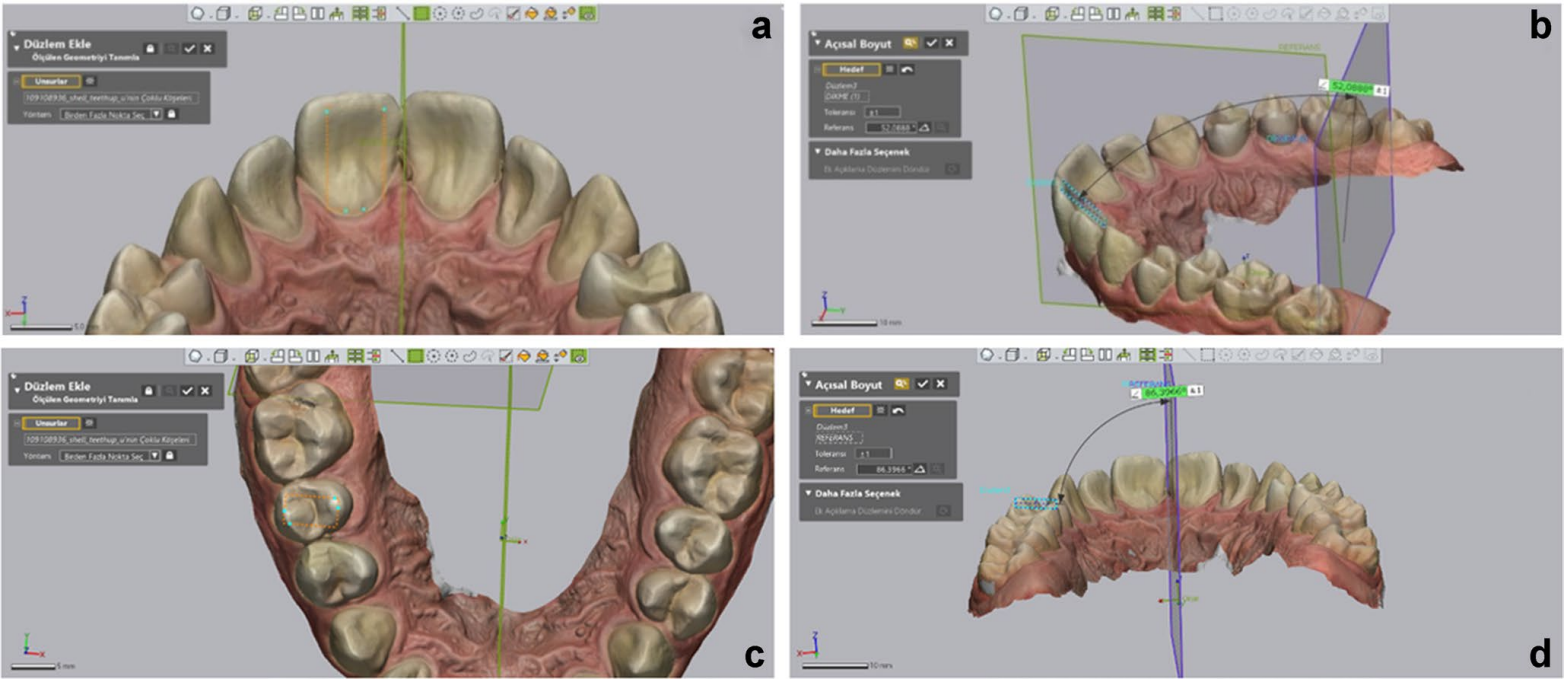
BLI measurement methodology. (**a**, **b**)Reference points and measurement of incisor, (**c**, **d**)Reference points and measurements of premolar

**BLI Measurement**: For incisors, a plane was defined using two key points on the palatal surface: the incisal edge and the deepest point of the cingulum. The angle between this plane and the MRP-90 was then measured. For premolars and molars, a plane was defined using four points selected at the cusp tips, and the angle between this plane and the MRP was measured (Fig. 6).

**Fig. 7:**
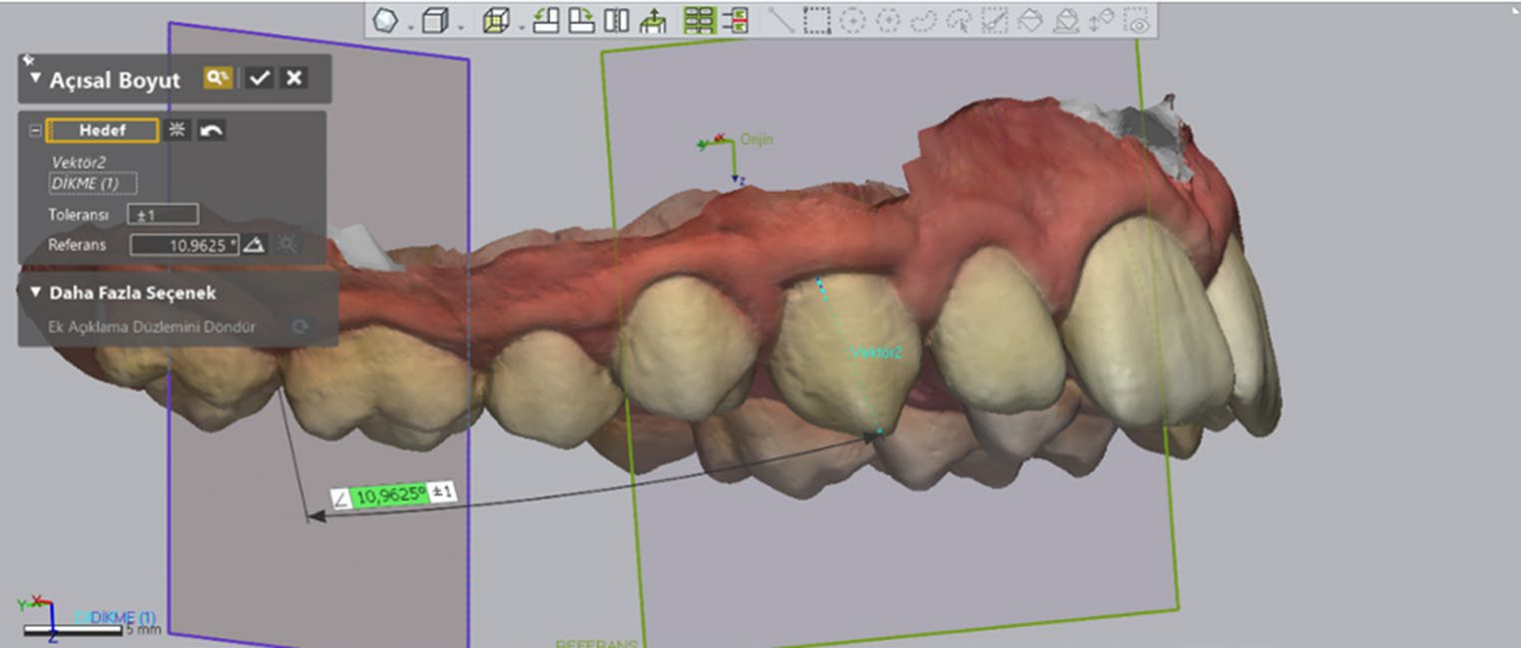
Angulation measurement methodology and reference point of canine

**Angulation measurement**: For each tooth, a vector was defined using the software’s “add vector” tool by selecting two points: the buccal midpoint of the incisal/occlusal edge and the midpoint of the cervical region. The angle between this vector and the MRP was measured for centrals and laterals, while the angle between this vector and the MRP-90 was measured for canines, premolars, and molars (Fig. 7).

**Fig. 8:**
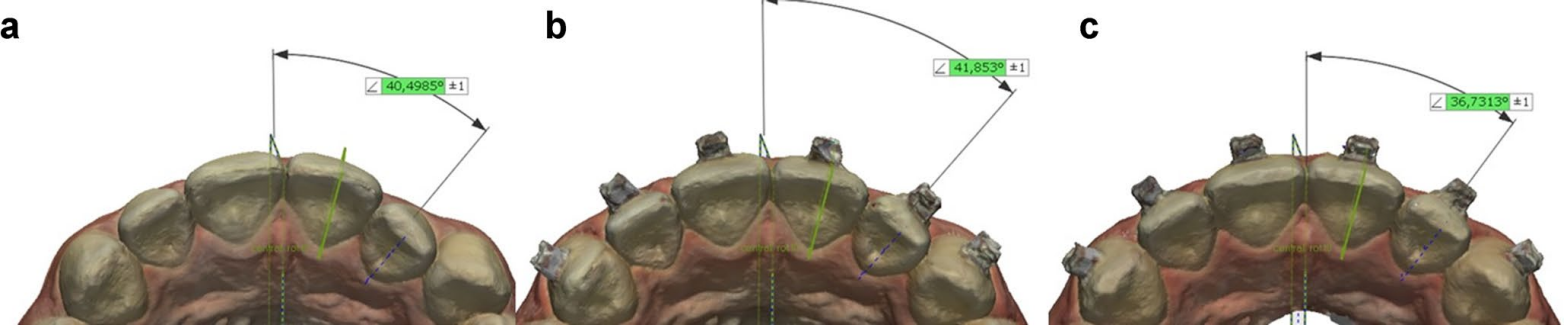
Total angular movement and sum of absolute movement (SAM) calculations. (**a**)T0, (**b**)T1, (**c**)T2

All measurements were repeated for T0, T1, and T2 time points. After the data were transferred to Microsoft Excel, the differences between the time points were calculated and recorded as absolute values. The values for incisors (11, 21, 12, 22), canines (13, 23), premolars (14, 24, 15, 25), and molars (16, 26) were averaged and categorized into groups. Net tooth movement was defined as the directional difference between T2 and T0 (T2–T0) and recorded as Net Movement (NET). To determine the total tooth movement over the 20-weeks period, the absolute differences between (T1-T0) and (T2-T1) were summed and recorded as the sum of absolute movement (SAM) (Fig. 8).

### Statistical analysis

Statistical analyses were performed in SPSS 26.0 (IBM, USA). Descriptive statistics were presented as mean ± SD for numerical variables and frequency (percentage). Normality was checked using Kolmogorov-Smirnov and Shapiro-Wilk tests. The differences between the groups were performed by one-way ANOVA with a Bonferroni post-hoc test. *p* < 0.05 values were considered a statistically significant result in all analyses.

## Results

For baseline crowding, there were no significant differences between the groups (*p* = 0.688), (Table 3). Similarly, baseline values of arch length, intercanine width, and intermolar width were comparable across groups (*p* > 0.240). At T2, and for T2–T0 changes, intercanine and intermolar widths differed significantly among groups (*p* < 0.05), while arch length did not (*p* > 0.274). Intercanine expansion was significantly greater in the Damon Q2 group than in the conventional group. Intermolar expansion was also significantly lower in the conventional group compared to both Damon groups. Arch length increased similarly in all groups (*p* > 0.05), (Table 4).

**Table 3:** Comparison of average maxillary malocclusion between the groups

Bracket System Groups	Maxillary Malocclusion			
	Mean	Min.	Max.	P*
Conventional	4.6 ± 0.77	3.2	5.8	*0.688*
Damon Ultima	4.5 ± 0.60	3.1	5.4	
Damon Q2	4.4 ± 0.56	3.4	5.2	

*: One-way *ANOVA* test

**Table 4:** The length measurements of different distances between the groups

Measurements	Groups	T0			T2			Mean Differences of T2-T0		
Width & Length										
		Mean	SD	p	Mean	SD	p	Mean	SD	p
Inter-canine	Conventional	33.33	2.96	0.24	34.76^a^	2.11	0.015*	1.44^a^	1.76	0.049*
	Damon Ultima	34.65	2.48		36.6^b^	2.25		1.95^ab^	2.90	
	Damon Q2	33.46	2.58		36.33^b^	1.86		2.88^b^	2.22	
Inter-molar	Conventional	45.47	2.69	0.49	45.9^a^	2.30	0.040*	0.43^a^	1.23	0.003*
	Damon Ultima	46.1	2.85		47.44^b^	2.56		1.35^b^	1.35	
	Damon Q2	46.9	2.53		47.98^b^	2.52		1.08^b^	1.20	
Arch Length	Conventional	64.45	3.4	0.27	66.46	3.06	0.274	2.02	1.32	0.72
	Damon Ultima	62.73	3.78		65.19	3.25		2.46	1.72	
	Damon Q2	63.96	3		66.49	2.25		2.53	3.06	

*: Significant at 0.05 level according to One-way *ANOVA* test; T0 and T2: time periods of measurements; *SD*: standard deviation

a,b: Different superscript letters (a and b) denote the significant pairwise comparison according to Bonferroni post-hoc test

For rotation movements, no significant group differences were found at baseline, T1, or T2 (*p* > 0.05), (Table 5a).

**Table 5:** **a.** Comparison of rotation, BLI, and angulation measurements between the groups

Measure-ments	Groups	T0			T1			T2		
		Mean	SD(±)	*p*	Mean	SD(±)	*p*	Mean	SD(±)	*p*
Rotations										
Incisors	*Conventional*	22.94	14.25	*0.475*	21.54	12.82	0.919	20.27	13.16	*0.154*
	*Damon Ultima*	24.99	14.15		21.51	14.03		18.93	13.68	
	*Damon Q2*	23.81	14.86		22.35	15.03		20.03	13.99	
Canines	*Conventional*	54.05	14.45	*0.518*	52.42	8.21	0.561	49.98	4.67	*0.660*
	*Damon Ultima*	50.43	7.98		51.49	4.95		49.90	5.15	
	*Damon Q2*	52.21	11.44		53.14	8.95		51.09	5.51	
Premolars	*Conventional*	72.36	7.02	*0.077*	71.16	4.84	0.617	72.54	4.22	*0.341*
	*Damon Ultima*	69.02	9.43		70.57	6.7		70.58	6.26	
	*Damon Q2*	68.48	8.62		70.35	6.08		71.38	4.75	
BLI										
Incisors	*Conventional*	47.21	11.37	*0.096*	48.38	9.11	0.003*	50.64	6.91	0.001*
	*Damon Ultima*	51.68	10.52		52.76	8.65		56.74	6.84	
	*Damon Q2*	52.23	12.28		52.57	8.89		55.47	6.29	
Canines	*Conventional*	53.37	11.85	*0.098*	55.35	8.84	0.163	58.95	6.94	*0.614*
	*Damon Ultima*	59.69	11.17		58.93	8.74		60.99	5.22	
	*Damon Q2*	53.92	12.10		55.92	10.36		60.02	8.02	
Premolars	*Conventional*	85.58	6.97	*0.995*	88.06	6.58	0.759	91.35	5.74	*0.175*
	*Damon Ultima*	85.76	7.89		88.2	7.41		94.16	6.49	
	*Damon Q2*	85.63	9.18		88.93	8.64		92.00	6.75	
Angulation										
Incisors	*Conventional*	5.37	3.87	*0.300*	3.64	2.28	0.226	3.12	2.34	*0.419*
	*Damon Ultima*	6.66	5.12		4.07	2.56		2.63	1.57	
	*Damon Q2*	6.60	4.45		4.32	2.81		2.83	1.64	
Canines	*Conventional*	7.23	5.82	*0.460*	4.39	3.16	0.540	3.65	2.35	*0.746*
	*Damon Ultima*	9.16	6.38		4.95	2.83		4.04	2.73	
	*Damon Q2*	7.62	5.22		5.16	3.21		3.58	2.06	
Premolars	*Conventional*	4.78	2.73	*0.322*	4.03	1.94	0.736	4.10	2.24	*0.051*
	*Damon Ultima*	5.58	3.57		4.12	2.49		3.09	2.23	
	*Damon Q2*	5.47	3.05		3.84	2.31		3.07	1.98	

*: Significant at 0.05 level according to One-way *ANOVA *test; T0 and T2:

time periods of measurements * SD: *

standard deviation; a, b: Different superscript letters Italicdenote the significant pairwise comparison according to Bonferroni post-hoc test

**Table 6:** **b.** Comparison of Net Movement and Sum of Absolute Movement measurements between the groups

Measure-ments	Groups	Net Movement T2-T0			Sum of Absolute Movement ( |T2-T1|+|T1-T0| )		
Rotations		Mean ± SD	SD (±)	*p*	Mean ± SD	SD	*p*
Incisors	*Conventional*	2.67	9.55	0.072	9.03	6.16	*0.669*
	*Damon Ultima*	6.06	9.39		10.11	7.38	
	*Damon Q2*	3.78	8.72		9.23	5.78	
Canines	*Conventional*	4.07	13.16	0.362	11.36	9.18	*0.269*
	*Damon Ultima*	0.53	8.66		8.37	5.21	
	*Damon Q2*	1.12	9.89		10.62	7.06	
Premolars	*Conventional*	-0.18	6.74	0.078	6.9	5.11	*0.426*
	*Damon Ultima*	-1.56	8.10		7.83	6.66	
	*Damon Q2*	-2.9	8.36		8.47	5.21	
BLI							
Incisors	*Conventional*	-3.43	9.23	0.34	9.26	6.62	*0.966*
	*Damon Ultima*	-5.06	8.42		9.45	3.87	
	*Damon Q2*	-3.24	8.77		9.15	5.88	
Canines	*Conventional*	-5.58	10.29	0.062	9.99	7.34	*0.638*
	*Damon Ultima*	-1.3	9.91		10.18	3.45	
	*Damon Q2*	-6.1	9.21		11.32	5.96	
Premolars	*Conventional*	-5.77^a^	4.59	0.003*	6.86 ^a^	3.53	0.014*
	*Damon Ultima*	-8.4^b^	5.07		9.28 ^b^	3.87	
	*Damon Q2*	-6.37^a^	6.44		8.43 ^ab^	4.97	
Angulation							
Incisors	*Conventional*	2.25^a^	2.86	0.003*	3.66 ^a^	2.72	0.021*
	*Damon Ultima*	4.03^b^	4.78		4.88 ^ab^	4.16	
	*Damon Q2*	3.77^b^	4.35		5.76 ^b^	4.51	
Canines	*Conventional*	3.58	5.19	0.442	6.02	5.32	*0.662*
	*Damon Ultima*	5.12	5.69		7.27	5.11	
	*Damon Q2*	4.04	5.07		6.69	4.69	
Premolars	*Conventional*	0.68^a^	3.48	0.001*	3.50 ^a^	2.27	0.014*
	*Damon Ultima*	2.49^b^	3.51		5.36 ^b^	3.64	
	*Damon Q2*	2.4^b^	3.51		4.61 ^ab^	2.98	

*: Significant at 0.05 level according to One-way *ANOVA* test; Net movement represents the directional change calculated as T2–T0, whereas the Sum of Absolute Movement (*SAM*) was calculated as |T2–T1| + |T1–T0|. Values are presented as mean and standard deviation (*SD*); a, b: Different superscript letters (a and b) denote the significant pairwise comparison according to Bonferroni post-hoc test

For BLI movements, incisor changes at T1 and T2 were significantly higher in the Damon groups than in the conventional group (*p* < 0.05). However, no group differences were detected at baseline. No significant differences were found for canine and premolar teeth groups at any time sections (*p* > 0.098), (Table 5a).

For angulation movements, no significant differences were found at the baseline, T1, T2 (*p* > 0.51), (Table 5a).

For rotational movements, no statistically significant intergroup differences were found in either net movement (T2–T0) or SAM values for incisors, canines, or premolars (*p* > 0.05). Although higher net rotational values were observed in the Damon Ultima group for incisors, these differences did not reach statistical significance (Table 6b).

For BLI, net movement and SAM values revealed no significant intergroup differences for incisors and canines (*p* > 0.05). However, for premolars, the Damon Ultima group exhibited significantly greater net BLI changes compared to other groups (*p* = 0.003), while SAM values were also significantly higher in the Damon Ultima group than in the conventional group (*p* = 0.014), (Table 6b).

For incisor angulation, net movement differed significantly among groups (*p* = 0.003). Both Damon groups demonstrated significantly higher net angulation changes compared to the conventional group. SAM values also revealed a significant intergroup difference (*p* = 0.021); however, this difference was primarily driven by the Damon Q2 group. For canine angulation, neither net movement nor SAM values differed significantly among groups (*p* > 0.44). For premolar angulation, the damon groups demonstrated significantly greater net angulation change compared to the conventional group (*p* < 0.001), while SAM values for the Damon Ultima group showed higher changes compared with the conventional group (*p* < 0.014), (Table 6b).

## Discussion

Self-ligating brackets are designed to reduce friction and potentially shorten orthodontic treatment time. Numerous studies have examined parameters such as crowding relief, arch expansion, and treatment duration [11–13]. However, limited research focuses on digital quantification of angular tooth movements [9, 14]. Yun et al. [9] analyzed tooth displacement via palatal rugae-based superimposition, while Stephens et al. [14] measured rotation in aligner cases using vector-based soft tissue reference points. In the present study, we utilized a three-point plane method to quantify movements, aiming to enhance precision and reproducibility. Additionally, all groups were treated using the same archwire sequence to control for this variable. This standardization allows the digital methodology to capture three-dimensional tooth movements more precisely and to isolate bracket-related effects more effectively.

The findings indicate that self-ligating systems produced greater changes in certain arch parameters compared to the conventional system. However, these changes did not result in clinically significant differences in the overall rate of tooth movement. Therefore, the null hypothesis was only partially rejected.

When the groups were compared at baseline, no significant differences were found in arch parameters or irregularity indices, except for molar angulation. The leveling rate may vary with increasing crowding [11]. Molars were excluded from the study to preserve randomization. Manufacturer-recommended molar tubes were used. Second molars were not included in the measurements because, after lower arch treatment began (2 weeks post-initiation), occlusion was raised using the upper second molars to avoid influencing tooth movement. As all patients received the same archwire sequence and clinical protocol, potential confounding variables were minimized by design. First molars were excluded from angular analyses because statistically significant baseline (T0) differences were detected between groups. Such initial discrepancies could have influenced the rate and pattern of correction, thereby compromising standardization and the comparability of angular changes across bracket systems.

In the present study, intercanine distance increased significantly in all groups, with the Damon Q2 group showing a greater increase than the conventional group, consistent with Damon’s findings [3]. This level of transverse expansion may extend beyond the physiologic limits of stability, highlighting the need for careful retention planning. However, some studies have reported conflicting intergroup comparisons [11, 15, 16]. Similarly, intermolar expansion was significantly greater in the Damon groups, aligning with previous literature [6, 11, 15]. Arch length also increased in all groups; however, the absence of significant intergroup differences contrasts with findings from earlier studies [11].

Intraoral scan timings were determined according to the archwire usage guidelines provided by Ormco. During the leveling phase, some teeth exhibited undesirable reactive movement, particularly in adjacent, supported units. This phenomenon was especially noted during the T1 assessments with 0.014” CuNiTi wires, where opposing directional movements were observed. As the primary aim of this study was to evaluate the rate of tooth movement, angular changes were recorded as absolute values and summed across each wire stage to calculate the total displacement, referred to as SAM. SAM values for the intervals T1–T0 and T2–T1 were used to represent total movement more accurately and to account for these reactive effects.

The amount of levelling that occurred between the beginning of treatment and the final recording (T2) was quantified by calculating the directional change as net movement (T2–T0). This approach allowed the resulting SAM values to be interpreted more clearly, as they reflect the cumulative magnitude of tooth movement throughout the levelling and aligning phase rather than a single end-point measurement. The SAM was considered clinically meaningful, as it captures total angular change regardless of direction. According to Newton’s third law of motion, every action has an equal and opposite reaction; in orthodontics, applying force to one tooth inevitably generates reactive forces in adjacent supporting teeth. These findings support the use of SAM over single-interval evaluations. As shown in Table 4, SAM may also serve as a useful reference when evaluating displacement patterns in clear aligner systems. Further studies are warranted to validate this approach.

Rotation values showed no significant differences between groups at either baseline (T0) or post-treatment (T2). Similarly, rotational SAM values were comparable across all groups. The Dentaurum Equilibrium (conventional group), has a greater mesiodistal width than the Damon systems, which theoretically increases rotational efficiency by extending the moment arm. However, differences in bracket friction levels may explain the observed similarity in rotational outcomes.

It should also be emphasized that differences observed between bracket systems cannot be attributed solely to whether a bracket is self-ligating or conventional. Biomechanically, rotational and angulation efficiency may also be influenced by factors such as frictional resistance, wire elasticity, and inter-bracket distance, the latter of which may itself be affected by the mesiodistal dimensions of the teeth. In our study, although the conventional bracket presented a greater mesiodistal width that would theoretically offer a longer moment arm for rotational correction, the clinical outcomes demonstrated the opposite trend. This suggests that bracket-specific frictional characteristics may override the geometric advantage of increased bracket width during the early alignment phase.

At T2, BLI values were significantly higher in the Damon groups than the conventional group, which may reflect the effect of the bracket design. Its rounded archwire system and base-in-torque bracket design may influence the elevated final BLI in the Damon Ultima group. Notably, this occurred despite lower built-in torque values in the Damon brackets. However, SAM values for BLI were statistically similar across all groups, suggesting that the overall inclination change was comparable. This may be attributed to similar levels of frictional resistance across systems. The so-called “lip bumper effect” of the perioral muscles, proposed by the manufacturer to limit incisor torque expression, was not supported by these findings.

Previous clinical studies have reported no significant difference in torque expression between conventional and Damon brackets for maxillary incisors [15, 17, 18]. In vitro analyses have similarly found that Damon systems do not produce statistically different torque outcomes compared to conventional brackets [19, 20]. However, our study did not include end-of-treatment assessments, limiting direct comparisons with these studies. Our study measured BLI digitally rather than torque values, limiting direct comparison. The higher BLI values observed in the Damon groups after rectangular wire use contrast with earlier torque-based findings, indicating a need for further investigation. Our study assessed BLI digitally, which is biomechanically related to torque but should not be interpreted as torque expression. The higher BLI values observed in the Damon groups after rectangular archwire insertion therefore reflect inclination changes rather than torque expression and should be considered within the limitations of early-stage alignment biomechanics.

Significant differences in angulation movement were observed between the groups at T2. The analysis revealed that the conventional group had the lowest SAM values, possibly due to increased friction, which is consistent with the reduced angulation correction observed. Although the Damon groups demonstrated greater overall movement across all teeth, these differences were insignificant for each parameter. The Dentaurum Equilibrium bracket system features a greater mesiodistal width, theoretically increasing the rotational moment for angulation correction [20]. However, the Damon systems exhibited more tooth movement despite this design feature, possibly due to increased wire elasticity and a greater inter-bracket distance. Additionally, the rapid correction observed in the Damon group may relate to reduced friction. The differences in built-in torque and bracket width across prescriptions did not fully translate into the expected clinical expression, indicating that friction, archwire elasticity, and inter-bracket distance may play a more dominant role than nominal prescription values.

The limitations of this study include the scarcity of prior research defining reproducible reference points or planes for evaluating torque, rotation, and angulation in fixed orthodontic treatment [9]. The reference planes used here were inspired by the only study on this topic and calibrated using digital methods, marking a step toward standardization. These planes were based on landmarks in the intraoral soft tissue, which may vary in position or volume over time despite their stability in the software. To achieve more reliable torque measurements in fixed appliances, future studies could consider using cone-beam computed tomography (CBCT) by aligning intraoral models with hard tissue structures. This approach would also allow for the inclusion of root positions in the analysis. However, CBCT-based methods may still face challenges in calibration and model alignment, and repeated imaging raises ethical concerns due to radiation exposure [21].

The SAM provides a comprehensive descriptor of cumulative tooth movement, it has not yet been formally validated against established orthodontic outcome measures.

Self-ligating brackets in our clinic are not used to manage skeletal transverse deficiencies; therefore, patients with skeletal maxillary constriction or an indication for RME were intentionally excluded. Patients with isolated dental crossbite involving one or a limited number of teeth were included, as these represented localized dentoalveolar discrepancies rather than true skeletal maxillary deficiency. Thus, the results of this study apply to dentoalveolar malocclusions without skeletal transverse problems and should not be extrapolated to patients requiring RME.

Finally, the 20-week follow-up reflects only the initial levelling and aligning phase; therefore, any interpretation of treatment efficiency must be limited to this early stage, and longer-term studies are required to fully evaluate the clinical impact of different bracket systems.

## Conclusions

Both Damon systems produced greater changes in intercanine and intermolar distances, despite using the same type of archwire across all groups; no significant effect was observed on arch length.

For rotational movement, no significant differences were found between bracket systems over the 20-week follow-up period.

For BLI and angulation in the relevant tooth groups, different amounts of tooth movement were observed between the bracket systems; however, the magnitude and clinical relevance of these changes should be interpreted with caution by clinicians.

The claimed benefits of reduced friction and treatment efficiency associated with self-ligating systems could not be validated.

Given the higher cost of self-ligating systems, the decision to use such brackets should be based on individual clinical judgment rather than expectations of superior tooth movement.

Additionally, reactive movements observed during the alignment phase may not occur in clear aligner treatments, suggesting that comparative studies between aligners and fixed appliances could offer further insight into the biomechanics of tooth movement.

## Data Availability

The datasets generated and/or analysed during the current study are not publicly available due to institutional data storage policies but are available from the corresponding author on reasonable request.

## Abbreviations

SAM: Sum of Absolute Movement
BLI: Bucco-Lingual Inclination
MRP: Middle Reference Plane
MRP-90: Perpendicular Plane to MRP
STL: Standard Triangle Language
ICC: Intraclass Correlation Coefficient
T0: Pre-treatment
T1: 12th week of treatment
T2: 20th week of treatment
